# Hippocampal neuronal metal ion imbalance related oxidative stress in a rat model of chronic aluminum exposure and neuroprotection of meloxicam

**DOI:** 10.1186/1744-9081-10-6

**Published:** 2014-03-11

**Authors:** Lijuan Yu, Rong Jiang, Qiang Su, Huarong Yu, Junqing Yang

**Affiliations:** 1 Department of Pharmacology, Chongqing Key Laboratory of Biochemistry and Molecular Pharmacology, Medical College Rd. No 1, Chongqing Medical University, Chongqing 400016, P. R. China; 2 Department of Stem Cells and Tissue Engineering, Chongqing Medical University, Chongqing 400016, P. R. China

**Keywords:** Neurodegeneration, Metal ion, Meloxicam, Aluminum overload, Oxidative stress

## Abstract

Neurodegenerative diseases remain a significant unresolved societal burden afflicting millions of people worldwide. Neurons in the brain are highly sensitive to oxidative stress, which can be induced by metal toxicity. In this paper, a chronic aluminum overload-induced model of neurodegeneration was used to investigate whether metal ions (Al, Fe, Mn, Cu and Zn)-related oxidative stress was involved in neurodegenerative mechanism and to identify the protective action of meloxicam against rat hippocampal neuronal injury. The metal ion contents, activity of superoxide dismutase (SOD), and content of malondialdehyde (MDA) were detected. The results showed that the spatial learning and memory (SLM) function was significantly impaired in chronic aluminum overload rats. Considerable karyopycnosis was observed in hippocampal neurons. The SOD activity was weakened and the MDA content increased both significantly. In the hippocampus, Al, Fe, Mn, Cu, and Zn contents increased by 184.1%, 186.1%, 884.2%, 199.4% and 149.2%, respectively. Meloxicam administration (without Al) had no effect compared with the control group, while meloxicam treatment with aluminum exposure significantly protected rats from SLM function impairment, neuron death, lower SOD activity, higher MDA content and brain metal ion imbalance. Our findings suggest that the cerebral metal ion imbalance-related oxidative stress is involved in mechanism of cerebral injury and neurodegeneration induced by chronic Al overload in rats, and that meloxicam protects neurons by reducing metal ion imbalance-related oxidative stress.

## Introduction

Neurodegenerative diseases (NDDs), including Alzheimer’s disease (AD), Parkinson’s disease (PD), Huntington’s disease (HD), Amyotrophic lateral sclerosis (ALS), Spinal muscular atrophy (SMA) and related neurological and psychiatric disorders, encompass a group of neurological disorders. Neurodegeneration can be described as loss of neuronal structure and function, and is manifested as loss of memory, cognition, movement or its control, and sensation [1]. For example, AD is characterized by memory loss and cognitive impairment [2], PD can cause cognitive impairment, including dementia and behavioral changes [3], and HD is manifested with dementia, involuntary motor activity, personality changes and cognitive impairment [4]. Though the current medical treatments have significantly improved the quality and length of life for NDD patients, NDDs remain a significant unresolved societal burden that afflicts millions of people worldwide.

NDDs are progressive, with reflective of increased neuron death. To date, the major mechanisms in pathogenic processes of NDDs include oxidative stress, protein aggregation, inflammation, blood brain barrier (BBB) disruption, and mitochondrial dysfunction. Oxidative stress is one major molecular mechanism responsible for the pathogenesis and progression of several NDDs [5]. Oxidative damage and mitochondrial dysfunction have been described in patients with AD, PD, HD, and ALS [6,7]. The misfolding and aggregation of specific proteins underlie many NDDs [8], and otherwise, neurotoxicant exposure may play a role in neurodegeneration [9]. Nevertheless, much research on neurodegeneration is fragmentary, leaving the mechanisms of NDDs unresolved.

The available treatments for NDDs are inadequate. The mainstay of treatment for AD is agents that inhibit the degradation of acetyl-choline in the synapse [10]. Current treatment options for PD include deep brain stimulation or increasing dopamine levels by providing a dopamine precursor, L-dopa, or dopamine agonists [11-13]. However, these treatments are effective at early stage in relieving symptoms, but ineffectiveness and long-term side-effects will gradually occur along with PD progression. Moreover, boosting autophagy can reduce protein accumulation and avoid toxicity due to protein aggregation in NDDs [14], and the utilization of stem cells may attenuate neurodegeneration [15]. However, the treatments are generally designed to alleviate symptoms, rather than reversing the progression of neurodegeneration. Thereby, a concerted inquiry is needed to decipher the mechanisms of NDDs, and accelerate the discovery of efficacious therapies.

Neurons in the brain are highly sensitive to oxidative stress, which can be induced by metal toxicity [16]. Previous experiments show that Al overload caused mouse brain damage and an increased expression of cyclooxygenase2 (COX2) [17]. Meloxicam as a selective COX2 inhibitor significantly protected mice from the Al-overload-caused brain damage [17]. In the present study, we established the neurodegeneration models of Wistar rats by long-term intragastric administration of aluminum gluconate [18,19], and investigated the changes of metal ion contents (Al, Fe, Mn, Cu, Zn), superoxide dismutase (SOD, an antioxidant enzyme) activity, and malondialdehyde (MDA, an oxidative stress biomarker) content. The aim of this study is to reveal whether the protective mechanism of meloxicam against rat hippocampal neuronal injury involves the reduction of the metal ion imbalance and oxidative stress.

## Materials and methods

### Animals

Experiments were approved by the Animal Laboratory Administrative Center and the Institutional Ethics Committee at Chongqing Medical University. Sixty male adult Wistar rats (obtained from Animal Laboratory Center of the University), weighing 200–250 g, were randomly and equally divided into 6 groups (*n* = 10): a control group, a model group, M-1 group, M-3 group, Al + M-1 group, and Al + M-3 group (M-1 and M-3 mean 1 and 3 mg · kg^−1^ meloxicam respectively).

### Chemicals

AlCl_3_ · 6H_2_O (Sinopharm Chemical Reagent Co., Ltd., China) and sodium gluconate (Beijing Qing Sheng Da Chemical Technology Co., Ltd., China) were of analytical grade. Meloxicam was purchased from Kunshan Rotam Reddy Pharmaceutical Co., Ltd (China). Aluminum gluconate solution (20 mg Al^3+^ · ml^−1^) was prepared on the day of experiments by adding 17.9 g of AlCl_3_ · 6H_2_O and 9.9 g of sodium gluconate into 100 ml of double distilled water (ddH_2_O) and then adjusted to about pH 6.0 [18,19].

### Establishment of animal models

The experiments were initiated after 3 days of acclimatization. The rats were treated by intragastric administration once a day, 5 d a week for 20 weeks as follows: the model group with 1 ml/100 g aluminum gluconate solution; the control group with the same volume of sodium gluconate solution; M-1 group and M-3 group with 1 and 3 mg.kg^−1^ meloxicam, respectively; Al + M-1 group and Al + M-3 group with 1 and 3 mg.kg^−1^ meloxicam respectively 30 min after administration of aluminum gluconate.

### Morris water maze test

At the second day after stopping aluminum gluconate administration, the spatial learning and memory (SLM) function was evaluated in a Morris water maze, following a reported method [20]. The water maintained at 24–25°C. In the learning stages, rats received 4 trials on each of 4 days. In each trial, a rat was placed into the water facing the pool wall, randomly from each of four starting positions. The trial was terminated and the latency was recorded when the rat found the platform within 180 s. Otherwise, the trial was terminated and the rat was led to the platform. On the fifth day, the rats received a probe trial in which the platform was removed. The rat was placed into the water as before to test its memory about the previous position of the platform.

### Histology

After the Morris water maze test, 3 rats from each group were perfused with heparinized saline (100 ml) to remove blood from the vasculature, and then with 4% paraformaldehyde in phosphate buffered saline (200 ml). The whole brain was then removed and stored in the same fixative. After paraffin embedding, 5-μm sections were obtained and stained with hematoxylin-eosin (H&E). Morphologic changes of hippocampal neurons were examined using light microscopy. For cell counting from H&E stained sections, 9 consecutive high power fields were sampled from the hippocampal CA1 subfield. Cells with a distinct nucleus and nucleolus were regarded as intact neurons. Neurons were counted using a microscope at 400× magnification. The extent of cell death was estimated by the count of intact cells divided by the total cell count [21].

### SOD activity assay

After Morris water maze test, 4 rat brains from each group were harvested. The hippocampi were homogenized with normal saline. Then SOD activity was detected using 0.05 ml of 1% homogenate (w/v) according to the manual of SOD assay kit (Jiancheng Bioengineering Ltd, Nanjing, China). The absorbance of samples at 550 nm was detected with a spectrophotometer (722, Shanghai Jinghua Technology Instrument Co., Ltd). The protein content was measured by the method of coomassie brilliant blue.

### MDA content assay

Hippocampal MDA content was detected according to the manual of the maleic dialdehyde assay kit (Jiancheng Bioengineering Ltd.). After Morris water maze test, brains were removed (*n* = 4). The hippocampi were homogenized with normal saline. MDA content was detected using 0.2 ml of 10% homogenate (w/v). The absorbance at 532 nm was detected with the spectrophotometer. The protein content was measured by the method of coomassie brilliant blue.

### Metal content detection

After Morris water maze test, 3 rat brains from each group were harvested. Each hippocampus was dissected and stored at −80°C until metal analysis. To detect metal contents in the brain, the hippocampus was weighed and homogenized in 2 ml of ddH_2_O. All homogenates were digested in 8 ml · g^−1^ wet brain of 25% tetraethyl ammonium hydroxide solution at 80°C for 24 h, and the mixture was then adjusted to a final volume of 10 ml with ddH_2_O and diluted 5 times before analysis. The tested metals were Al, Fe, Mn, Cu, and Zn and were analyzed by inductively coupled plasma-atomic emission spectrometry (ICP-AES). To avoid interference of metal ions, one-off plastic test tubes were used during procedures.

### Statistical analysis

The results were expressed as mean ± standard deviation (SD) and processed on SPSS 12.0 (SPSS Inc. Chicago, US). Within-group variances were compared by Dunnett’s t-test.

## Results

### Changes of SLM function

The time taken to find the platform (latency) in the model group was significantly longer compared with the control group. The latency in both Al + M-1 and Al + M-3 groups was significantly shortened in a dose-dependent manner compared with the model group. There was no significant difference among the control, M-1 and M-3 groups (Table 1).

**Table 1:** Changes of spatial learning and memory function of chronic aluminum overload rats (mean ± SD, n = 10)

**Group**	**Latency (s)**				
	**Day 1**	**Day 2**	**Day 3**	**Day 4**	**Day 5**
Control group	100.21 ± 9.38	75.32 ± 9.51	42.16 ± 7.24	20.31 ± 4.88	14.24 ± 3.38
M-3 group	96.28 ± 8.51	65.37 ± 7.32	33.46 ± 6.27	19.34 ± 4.08	12.04 ± 3.22
M-1 group	104.36 ± 12.14	72.52 ± 9.11	40.66 ± 7.16	23.11 ± 4.25	15.43 ± 4.39
Model group	158.42 ± 24.03^**^	102.56 ± 11.36^**^	70.53 ± 7.58^**^	53.16 ± 6.24^**^	45.14 ± 6.13^**^
Al + M-3 group	99.33 ± 9.18^##^	69.51 ± 8.17^##^	38.69 ± 5.11^##a^	20.14 ± 4.22^##a^	16.14 ± 4.10^##a^
Al + M-1 group	114.18 ± 8.68^##^	79.33 ± 9.41^##^	53.18 ± 7.59^##^	36.17 ± 4.56^#^	24.23 ± 3.62^#^

^**^P < 0.01, vs. control group; ^#^P < 0.05, ^##^P < 0.01, vs. model group; ^a^P < 0.05, vs. Al + M-1 group; M-3 group: meloxicam 3 mg · kg^−1^ group; M-1 group: meloxicam 1 mg · kg^−1^ group; Al + M-3 group: Al + meloxicam 3 mg · kg^−1^ group; Al + M-1 group: Al + meloxicam 1 mg · kg^−1^ group.

### Changes of neuronal pathomorphology

**Figure 1:**
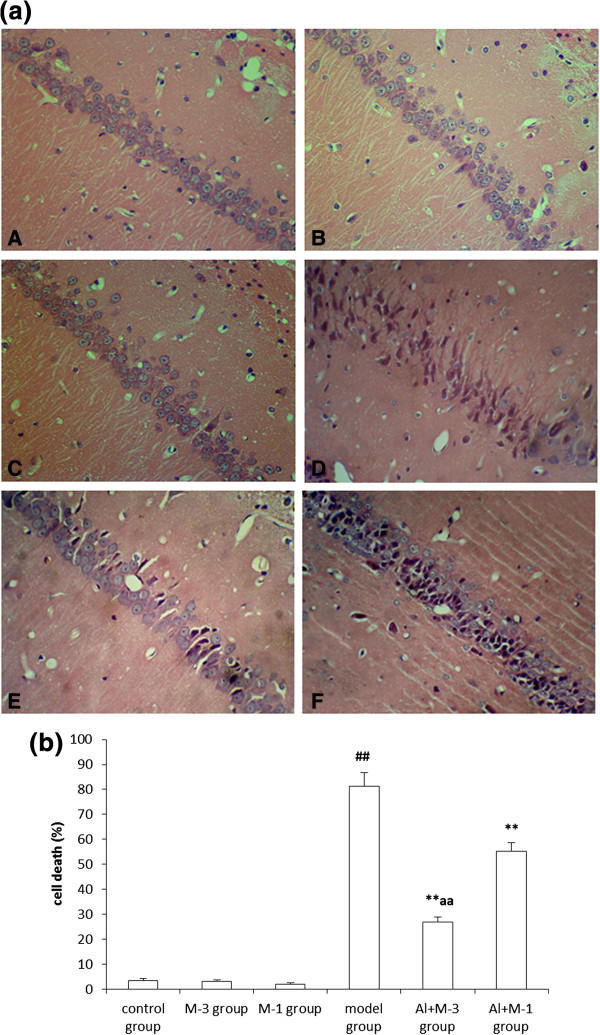
**Changes of neuronal pathomorphology in chronic aluminum overload rat hippocampus. a.** Neuronal pathomorphology in chronic aluminum overload rat hippocampus (HE × 400). A: Control group; B: Meloxicam 3 mg kg^−1^ group; C: Meloxicam 1 mg kg^−1^ group; D: Model group; E: Al + Meloxicam 3 mg kg^−1^ group; F: Al + Meloxicam 1 mg kg^−1^ group. **b.** Group data showing the effect of meloxicam on the cell death rate. ^##^P < 0.01, vs. control group; **P < 0.01, vs. model group; ^aa^P < 0.01, vs. Al + M-1 group; Al + M-3 group: Al + meloxicam 3 mg kg^−1^ group; Al + M-1 group: Al + meloxicam 1 mg kg^−1^ group (mean ± SD, n = 3).

The hippocampal neurons were in distinct and regular structure, and arranged densely and clearly in the control, M-1 and M-3 groups. In contrast, the model group revealed significant injuries including remarkable cell loss and karyopycnosis in hippocampal neurons. Dead and dying cells in the injured hippocampi displayed necrosis, karyopycnosis and irregular contours. The cell loss and karyopycnosis were significantly diminished in the Al + M-3 group in particular and the Al + M-1 group (Figure 1a). Quantification of remaining, the Al + M-3 group (P < 0.01) and the Al + M-1 group (P < 0.01) exhibited 27.96% and 54.05% reduction in cell death, respectively compared with the model group. There was significant difference between the Al + M-3 group and the Al + M-1 group (P < 0.01), but no significant difference among the control, M-1 and M-3 groups (Figure 1b).

### Changes of SOD activity

SOD activity in chronic aluminum overload rats distinctly decreased compared with the control group. Meloxicam administration significantly reversed the decrease of SOD activity caused by aluminum overload, especially in the Al + M-3 group. And there was no significant difference among the control, M-1 and M-3 groups (Table 2).

**Table 2:** Changes of SOD activity of chronic aluminum overload rat hippocampus (mean ± SD, n = 4)

**Group**	**SOD (U · mg**^**−1**^**)**
Control group	10.84 ± 1.75
meloxicam 3 mg · kg^−1^ group	11.03 ± 1.54
meloxicam 1 mg · kg^−1^ group	10.22 ± 1.37
Model group	8.09 ± 0.72^*^
Al + meloxicam 3 mg · kg^−1^ group	14.99 ± 1.40^#b^
Al + meloxicam 1 mg · kg^−1^ group	9.81 ± 0.97^#^

^*^P < 0.05, vs. control group; ^#^P <0.05, vs. model group; ^b^P < 0.01, vs. Al + meloxicam 1 mg · kg^−1^ group.

SOD: superoxide dismutase.

U · mg^−1^: Unit per mg of protein.

### Changes of MDA content

MDA content in the model group significantly increased compared to the control group. Meloxicam administration significantly blunted the increase of MDA content in chronic aluminum overload rats. There was no significant difference among the control, M-1 and M-3 groups (Table 3).

**Table 3:** Changes of MDA content of chronic aluminum overload rat hippocampus (mean ± SD, n = 4)

**Group**	**MDA (nmol · mg**^**−1**^**)**
Control group	0.57 ± 0.07
meloxicam 3 mg · kg^−1^ group	0.54 ± 0.08
meloxicam 1 mg · kg^−1^ group	0.56 ± 0.05
Model group	1.32 ± 0.22^*^
Al + meloxicam 3 mg · kg^−1^ group	0.41 ± 0.02^#b^
Al + meloxicam 1 mg · kg^−1^ group	0.60 ± 0.06^#^

^*^P < 0.05, vs. control group; ^#^P <0.05, vs. model group; ^b^P < 0.01, vs. Al + meloxicam 1 mg · kg^−1^ group.

MDA: malondialdehyde.

nmol · mg^−1^: nmol per mg of protein.

### Changes of metal ion contents

#### Al

**Figure 2:**
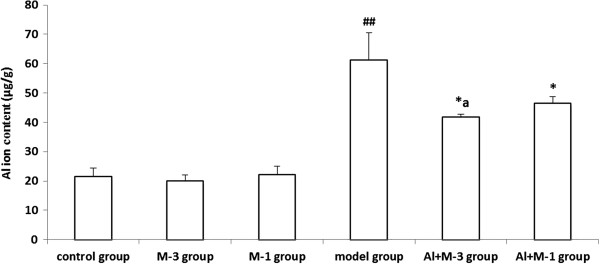
**Changes of aluminum content of chronic aluminum overload rat hippocampus (mean ± SD, n = 3). **^##^P < 0.01, vs. control group; *P < 0.05, vs. model group; ^a^P < 0.05, vs. Al + M-1 group; M-3 group: meloxicam 3 mg · kg^−1^ group; M-1 group: meloxicam 1 mg · kg^−1^ group; Al + M-3 group: Al + meloxicam 3 mg · kg^−1^ group; Al + M-1 group: Al + meloxicam 1 mg · kg^−1^ group. μg/g: μg per g of wet weight tissue.

Al content in the model group was significantly increased by 184.1% compared with the control group. There was no significant difference among the control, M-1 and M-3 groups. Al content significantly decreased in the Al + M-3 group in particular and the Al + M-1 group compared with the model group (Figure 2).

#### Fe

**Figure 3:**
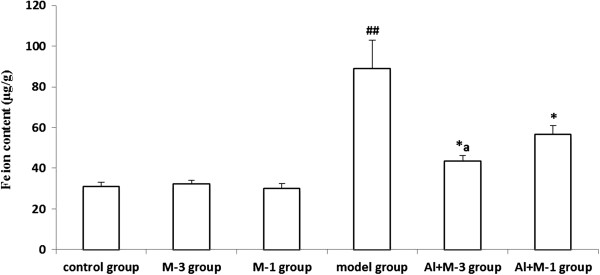
**Changes of iron content of chronic aluminum overload rat hippocampus (mean ± SD, n = 3). **^##^P < 0.01, vs. control group; ^*^P < 0.05, vs. model group; ^a^P < 0.05, vs. Al + M-1 group; M-3 group: meloxicam 3 mg · kg^−1^ group; M-1 group: meloxicam 1 mg · kg^−1^ group; Al + M-3 group: Al + meloxicam 3 mg · kg^−1^ group; Al + M-1 group: Al + meloxicam 1 mg · kg^−1^ group. μg/g: μg per g of wet weight tissue.

Fe content in the rat hippocampus was the highest among the tested metals (Al, Fe, Mn, Cu and Zn) in the control group. Fe contents of the M-1 and the M-3 groups were equal to that of the control group. Fe content in the model group was significantly increased by 186.1% compared with the control group. Meloxicam administration significantly decreased the Fe contents in the Al + M-3 and Al + M-1 groups, and there was significant difference between the two groups (Figure 3).

#### Mn

**Figure 4:**
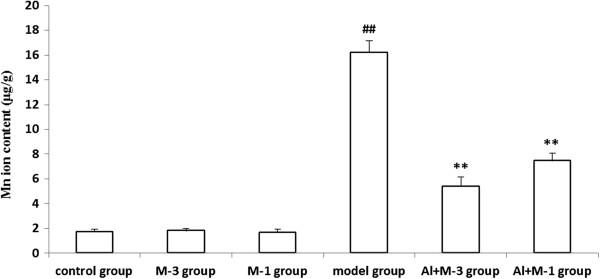
**Changes of manganese content of chronic aluminum overload rat hippocampus (mean ± SD, n = 3). **^##^P < 0.01, vs. control group; **P < 0.01, vs. model group; M-3 group: meloxicam 3 mg · kg^−1^ group; M-1 group: meloxicam 1 mg · kg^−1^ group; Al + M-3 group: Al + meloxicam 3 mg · kg^−1^ group; Al + M-1 group: Al + meloxicam 1 mg · kg^−1^ group. μg/g: μg per g of wet weight tissue.

Mn content in the rat hippocampus was the lowest among the tested metals. Mn content of the model group increased by 884.2% compared with control group. Considering the increasing ratio, Mn content increased the most severely. There was no significant difference among the control, M-1 and M-3 groups. Mn contents of the Al + M-3 group in particular and the Al + M-1 group significantly decreased compared with the model group (Figure 4).

#### Cu

**Figure 5:**
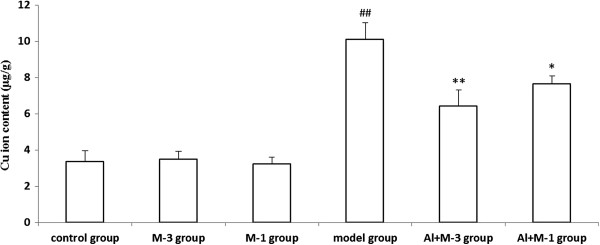
**Changes of copper content of chronic aluminum overload rat hippocampus (mean ± SD,n = 3). **^##^P < 0.01, vs. control group; *P < 0.05, **P < 0.01, vs. model group; M-3 group: meloxicam 3 mg · kg^−1^ group; M-1 group: meloxicam 1 mg · kg^−1^ group; Al + M-3 group: Al + meloxicam 3 mg · kg^−1^ group; Al + M-1 group: Al + meloxicam 1 mg · kg^−1^ group. μg/g: μg per g of wet weight tissue.

Cu content of the model group significantly increased by 199.4% compared with the control group. Cu contents of the M-1 and M-3 groups were equal to that of the control group. Meloxicam administration significantly decreased the Cu contents in the Al + M-3 and Al + M-1 groups, and there was significant difference between the two groups (Figure 5).

#### Zn

**Figure 6:**
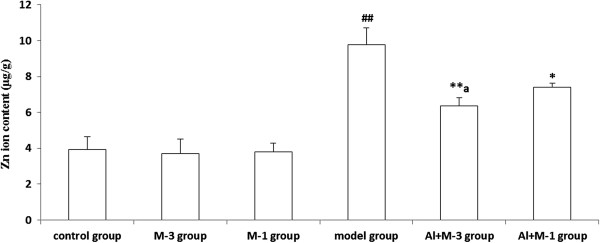
**Changes of zinc content of chronic aluminum overload rat hippocampus (mean ± SD, n = 3). **^##^P < 0.01, vs. control group; *P < 0.05, **P < 0.01, vs. model group; ^a^P < 0.05, vs. Al + M-1 group; M-3 group: meloxicam 3 mg · kg^−1^ group; M-1 group: meloxicam 1 mg · kg^−1^ group; Al + M-3 group: Al + meloxicam 3 mg · kg^−1^ group; Al + M-1 group: Al + meloxicam 1 mg · kg^−1^ group. μg/g: μg per g of wet weight tissue.

Zn content of the model group significantly increased by 149.2% compared with the control group. There was no significant difference among the control, M-1 and M-3 groups. Zn contents of the Al + M-3 group in particular and the Al + M-1 group significantly decreased (Figure 6).

## Discussion

Metal ions are required for maintaining the functions of many proteins and proper metal ion balance in the brain is significant for normal cognitive function [22]. Thus, metal ions have received exponentially increasing interest. Growing evidence has been collected on the relationship between metal ions and the development of neurological disorders, such as metal-protein association inducing protein aggregation and metal-catalyzed protein oxidation inducing protein damage and/or generation of reactive oxygen species (ROS) [23,24]. Metals such as Al, Fe, Cu, and Zn were dysregulated in AD brain tissue to create a pro-oxidative environment [25-29]. In the frontal cortex of young and aged rats fed with AlCl_3_, the Al, Fe and Zn contents significantly increased and Al may be linked with alteration in neurobehavioral activity [30]. The multifunctional metal-ion chelators as a potential treatment for metal-promoted neurodegenerative diseases (MpND) has attracted much attention and showed promise of disease-modifying [31-34].

Al as an important neurotoxin has been investigated extensively both in vitro and in vivo, and is associated with cognitive dysfunction and various mental diseases. Recent neuropathological, biochemical, and epidemiological studies suggest that Al contributes to the progression of several NDDs, including AD, and PD, but the precise mechanism has not been clarified yet [30,35-37]. Intracerebroventricular (icv) injection of trace AlCl_3_ into mice will result in neurodegeneration and learning/memory disorders [38]. However, oral ingestion is the main form of Al exposure in clinic. Because the icv animal models do not much resemble that from oral ingestion of Al, several scientists hold that the icv AlCl_3_ injection model does not strictly speak a neurodegeneration model. In the present study, we established neurodegenerative models by intragastric administration of aluminum gluconate (200 mg Al^3+^ · Kg^−1^, once a day, 5 d a week, for 20 weeks) [18,19]. The results showed that the SLM function was significantly impaired and significant karyopycnosis of hippocampal neurons was observed in the model group compared with the control group.

Al neurotoxicity may be related to the integrity and permeability of BBB [39]. Al can induce apoptosis in rat hippocampal cells through the down-regulation of bcl-2 mRNA expression and the up-regulation of bax mRNA expression [40]. Al may also be involved in the aggregation of Aβ peptides, inducing Aβ peptides into the β-sheet structure and facilitating iron-mediated oxidative reactions [41]. Neurodegeneration caused by aluminum overload was associated with an imbalance in metal ion levels in the brain. Metal dyshomeostasis is linked in protein misfolding and may contribute to oxidative stress and neuronal damage. The presence of Al might change the contents of endogenous trace metals [42].

Iron as an important trace element is essential for neuron development since it is required for various physiological events, including mitochondrial respiration, oxygen transport and DNA synthesis [43]. However, iron contributes to oxidative stress through Fenton reaction, leading to damages in DNA, proteins and membrane [44,45]. Iron imbalance is a precursor to the neurodegenerative processes leading to AD [46], and quantification of brain iron content can be an effective marker for early diagnosis of AD [47]. Iron accumulation may contribute to protein aggregation and neuronal death in PD patients [48]. Excessive iron would induce cell injury by reacting with H_2_O_2_ to produce hydroxyl radical (OH^−^), superoxide anions (O_2_^−^), and ROS [49]. Another hypothesis states that iron-mediated free radical production contributes to BBB opening to cause neuronal damage [50]. In our study, the iron content in the model group was significantly higher compared with the control group, and iron content was the highest among the tested metals, implying that iron overload in hippocampus may play an important role in the occurrence of neuron damage.

Other transitional metals such as Mn, Cu, and Zn are essential enzyme cofactors required for numerous cellular processes, but their abnormal accumulation in the brain will lead to neurotoxicity [22]. Mn has long been known to cause neurological disorders similar to PD. Mn might result in movement abnormalities in PD patients [51]. The present study revealed that Mn content in hippocampus of the model group was 8.8 times (the highest ratio) higher compared with the control group. The mechanism of Mn-induced neurotoxicity has not been fully elucidated, but an established mechanism is correlated with attenuated uptake of glutamate (GSH) [52]. Mn can reduce brain glutathione level, likely reflecting oxidative stress [53], and might lead to mitochondrial dysfunction and trigger apoptotic-like neuronal death [54]. These studies indicate that the obvious increase of Mn content in hippocampus may play a key role in the mechanism of chronic Al-induced brain damage and neural degeneration.

Cu which is released at the synaptic cleft is an important structural cofactor in a series of biochemical processes with a narrow-range of optimal content [55]. The knowledge of Cu homeostasis has become increasingly important in clinical medicine, as it can be involved in the pathogenesis of NDDs such as AD [56-59]. The mechanism may be that Cu affects the degradation and aggregation of Aβ in AD [60,61]. We found that Cu content significantly increased after 20-week administration of aluminum gluconate, and this may be a reason for the SLM function impairment and neuron death.

Zn, essential for human health in trace amounts, is co-released with GSH and the significance of Zn signaling is gradually recognized [62]. Hippocampal pyramidal neurons are vulnerable to brain injury, while Zn entry may enhance this vulnerability [63]. Zn has been implicated in AD and PD. Excessive Zn translocation might be a molecular trigger of the cellular apoptosis [64,65]. In our experiments, the hippocampus of model rats showed Zn accumulation, and we thought that Zn is also involved in the occurrence of brain injury.

Neurons in brain are highly sensitive to oxidative stress. Metal toxicity is a problem leading to oxidative stress. Superoxide radicals can also create further oxidative stress by metal-catalyzed reactions [16]. SOD converts superoxide to H_2_O_2_ and oxygen. SODs are the most important antioxidant enzymes in the antioxidant defense system [66]. MDA is an end-product of lipid peroxidation and an excellent marker for degeneration of neurons [67]. Besides, metal ion contents in hippocampus of the model group significantly increased compared with the control group. The hippocampal SOD activity was weakened and MDA content increased both significantly in the model group. The results might further confirm the hypothesis that imbalance of cerebral metal ion is involved in occurrence of oxidative stress.

Moreover, meloxicam could significantly suppress metal ion elevation and prevent hippocampal neuron injury in aluminum overload rats. Reportedly, COX-2-induced synthesis of prostaglandins (PGs) was associated with chronic inflammation [68,69], causing oxidative stress. Our previous study showed that chronic aluminum overload significantly elevated COX2 mRNA and protein expressions [18]. These results suggest that as a selective COX2 inhibitor, meloxicam might alleviate oxidative stress damage to the brain by inhibiting COX2 activity, relieving inflammation and reducing metal ion imbalance. It may be involved in the neuroprotective mechanism of meloxicam against rat hippocampal neuronal injury following chronic aluminum overload.

In conclusion, we provide evidence that metal ion imbalance may contribute significantly to hippocampal injury caused by exposure to aluminum. Meloxicam was neuroprotective by decreasing COX2 expression and was associated with inhibition of oxidative stress. Clearly, further studies are necessary to clarify the neuroprotective mechanisms of meloxicam after exposure to aluminum.

## Competing interest

The authors declare no conflicts of interest/disclosures.

## Authors’ contributions

LY contributed to the data collection, analyzed and interpreted the data, and wrote the manuscript. RJ and QS collected the data and conducted the experiments. HY and JY participated in the concept and design of the study and edited the manuscript. All authors read and approved the final manuscript.
